# Phages of non-dairy lactococci: isolation and characterization of ΦL47, a phage infecting the grass isolate *Lactococcus lactis* ssp. *cremoris* DPC6860

**DOI:** 10.3389/fmicb.2013.00417

**Published:** 2014-01-13

**Authors:** Daniel Cavanagh, Caitriona M. Guinane, Horst Neve, Aidan Coffey, R. Paul Ross, Gerald F. Fitzgerald, Olivia McAuliffe

**Affiliations:** ^1^Department of Food Biosciences, Teagasc Food Research CentreFermoy, Ireland; ^2^Department of Microbiology, University College CorkCo. Cork, Ireland; ^3^Department of Microbiology and Biotechnology, Max Rubner-Institut, Federal Research Institute of Nutrition and FoodKiel, Germany; ^4^Department of Biological Sciences, Cork Institute of TechnologyCo. Cork, Ireland

**Keywords:** *Lactococcus lactis*, non-dairy, phage, tail fiber, genome

## Abstract

Lactococci isolated from non-dairy sources have been found to possess enhanced metabolic activity when compared to dairy strains. These capabilities may be harnessed through the use of these strains as starter or adjunct cultures to produce more diverse flavor profiles in cheese and other dairy products. To understand the interactions between these organisms and the phages that infect them, a number of phages were isolated against lactococcal strains of non-dairy origin. One such phage, ΦL47, was isolated from a sewage sample using the grass isolate *L. lactis* ssp. *cremoris* DPC6860 as a host. Visualization of phage virions by transmission electron microscopy established that this phage belongs to the family *Siphoviridae* and possesses a long tail fiber, previously unseen in dairy lactococcal phages. Determination of the lytic spectrum revealed a broader than expected host range, with ΦL47 capable of infecting 4 industrial dairy strains, including ML8, HP and 310, and 3 additional non-dairy isolates. Whole genome sequencing of ΦL47 revealed a dsDNA genome of 128, 546 bp, making it the largest sequenced lactococcal phage to date. In total, 190 open reading frames (ORFs) were identified, and comparative analysis revealed that the predicted products of 117 of these ORFs shared greater than 50% amino acid identity with those of *L. lactis* phage Φ949, a phage isolated from cheese whey. Despite their different ecological niches, the genomic content and organization of ΦL47 and Φ949 are quite similar, with both containing 4 gene clusters oriented in different transcriptional directions. Other features that distinguish ΦL47 from Φ949 and other lactococcal phages, in addition to the presence of the tail fiber and the genome length, include a low GC content (32.5%) and a high number of predicted tRNA genes (8). Comparative genome analysis supports the conclusion that ΦL47 is a new member of the 949 lactococcal phage group which currently includes the dairy Φ949.

## Introduction

Cultures of lactic acid bacteria (LAB) used in cheese manufacture play a pivotal role in the formation of cheese flavor (Urbach, 1995). While the limited numbers of established dairy cultures in use have greatly reduced inconsistencies in cheese quality, it can be at the expense of cheese flavor. LAB isolated from non-dairy environments, such as plant material, often exhibit enhanced metabolic capabilities when compared to those of dairy origin and have been shown to contribute to a more diverse flavor profile in the cheese (Ayad et al., 1999, 2000; Morales et al., 2003). In addition to their role in flavor enhancement, cultures of non-dairy origin have also been shown to be naturally insensitive to bacteriophages which infect industrial strains (Ayad et al., 2000). Phage infection is the single largest cause of industrial milk fermentation problems, negatively impacting on the consistency of cheese quality and resulting in large economic losses (Coffey and Ross, 2002). Non-dairy lactococcal strains could potentially be exploited for use in cheese culture rotations as phage insensitive strains and may well reduce the negative consequences of phage infection within a production plant. However, given the high evolution rate of phages, it is highly likely that over time, phages would emerge to threaten these bacterial strains.

All lactococcal phages isolated to date are members of the *Caudovirales* order which comprises of three families: *Siphoviridae* (long, non-contractile tails), *Myoviridae* (long, contractile tails) and *Podoviridae* (short tails) [for review see Veesler and Cambillau (2011)]. Phage classification systems have been utilized in the design of anti-phage strategies to prevent phages amplifying to high titers in manufacturing plants (Jarvis et al., 1991). DNA hybridization assays and comparative genomic analysis coupled with visualization of virions has led to the division of lactococcal phages into one of ten groups (Deveau et al., 2006). Of these phage groups, the c2, 936 and P335 groups are predominantly associated with failed dairy fermentations and are frequently isolated from cheese manufacturing plants (Moineau et al., 1992; Murphy et al., 2013). Currently within the NCBI database, there are complete genome sequences for over 30 lactococcal bacteriophages. Phages classified within the same group can share a large degree of genome similarity as observed within the c2 and 936 groups (Lubbers et al., 1995; Chopin et al., 2001; Crutz-Le Coq et al., 2002). However, within the P335 group, there exists a high proportion of genetic diversity attributed to the presence of both temperate and virulent bacteriophages classified within this group (Labrie and Moineau, 2007; Garneau et al., 2008). Collectively, these groups account for the majority of sequenced lactococcal phage genomes, ranging in size from 22 to 40 kb. Larger lactococcal phages do also exist, with Φ949 at 114.8 kb being the largest sequenced lactococcal phage to date (Samson and Moineau, 2010). With the elucidation of the Φ949 genome, a genomic sequence is available for at least one member of each phage group.

The use of a wide range of different bacterial strains possessing an industrially important phenotype has presumably contributed to the diversity of lactococcal phages. Genome analysis of the rarer uncommon lactococcal phages has shed further light on this diversity. Comparative analysis of the structural proteins of Φ1358, show a high degree of similarity to 2 *Listeria monocytogenes* phages. Moreover this phage was also found to possess an uncommonly high %GC content of 51 (Dupuis and Moineau, 2010). Recent analysis of the Φ1706 genome, revealed that 22 ORFs shared similarities with proteins of Firmicutes, typically found in the human gut (Garneau et al., 2008). The ability of this phage to infect lactococci was attributed to the acquisition of a 4 gene module, allowing for host recognition of lactococcal cells (Garneau et al., 2008). Genomic analysis of the ΦKSY1 genome has suggested the exchange of genetic material between bacteria and phages from different environments (Chopin et al., 2007). ΦQ54 was found to possess a different modular configuration, thought to be derived from recombination events with 936 and c2 type phages (Fortier et al., 2006). It is probable that these rarer phages arose from recombination events with other lactococcal phages and phages infecting other Gram-positive bacteria (Dupuis and Moineau, 2010). Moreover, these phages appear to be less suited to thrive in milk fermentations in contrast to phages of the more common lactococcal phage groups which possess rapid reproduction rates believed to be driven by evolutionary pressure (Ferguson and Coombs, 2000; Dupuis and Moineau, 2010).

In this study we describe the isolation and characterization of ΦL47, a large lytic phage which infects the non-dairy isolate *L. lactis* ssp. *cremoris* DPC6860. We also report the complete genome sequence of ΦL47 which, to our knowledge, is the largest lactococcal phage reported to date. Due to the emergence of non-dairy lactococci as dairy cultures with enhanced flavor-forming activity, sufficient data needs to be generated with regards to these cultures and their phage resistance if they are to be successfully utilized in dairy processing. Therefore, the objective of this study was to provide a better understanding of phages of lactococci from non-dairy origins at a phenotypic and genomic level, thus offering further insight into phage-host interactions.

## Materials and methods

### Bacterial strains, bacteriophage and culture conditions

Dairy and non-dairy *Lactococcus* strains were supplied by the TFRC-Moorepark culture collection (Teagasc Food Research Centre, Moorepark, Ireland). *L. lactis* ssp. *cremoris* DPC6860 was previously isolated from grass (D. Cavanagh, unpublished) and characterized by 16S rRNA analysis as described by Alander et al. (1999). All lactococcal strains used in this study were cultured in M17 (Oxoid, Hampshire, England) media containing 0.5% lactose (wt./vol.) (VWR, Leuven, Belgium) (LM17) at 30°C for 18–24 h under aerobic conditions. Double-strength M17 broth, used for phage enrichment of sewage samples, was prepared by doubling the amount of dry M17 media and reconstituting it in the same amount of distilled water as the 1X media with the addition of 1% lactose (wt./vol.). Soft agar overlays and solid agar medium contained 0.75% and 1.5% agar, respectively. Lactococcal phages ΦKSY1, Φc2, ΦbIL170 and ΦP008 were originally obtained from the Felix d'Hérelle Reference Center for Bacterial Viruses (GREB, Pavillon de Médecine Dentaire Université Laval, QC, Canada**)** while phages Φ712, ΦHP, ΦebI and ΦML3 were obtained from the UCC culture collection (University College Cork, Cork, Ireland). Phages were propagated using their respective hosts at 30°C in M17 media containing 0.5% glucose (wt./vol.) (Sigma-Aldrich, Dublin, Ireland) and 10 mM CaCl_2_.

### Spot assay

Bacterial infection by phages was assessed using spot plate assays with phage titers of 10^8^ PFU/mL. Briefly, 10 μ L of phage lysate was spotted onto an LM17 soft agar overlay containing 10 mM CaCl_2_ and inoculated with 1 × 10^8^ CFU/mL of the host organism. Spot plates were allowed to dry before incubation aerobically, at 30°C for 24 h.

### Phage isolation and propagation

Raw sewage samples were collected from the sewage treatment facility in Mitchelstown, Co. Cork, Ireland. Phage isolation was conducted as described previously (Alemayehu et al., 2009). Individual plaques isolated following this method underwent 3 successive rounds of plaque purification, to ensure that a pure phage was isolated. Briefly, a single plaque was aseptically removed from an overlay plate using a sterile 1 mL pipette tip and added to 5 mL of mid-log phase host, containing 10 mM CaCl_2_. Following overnight incubation at 30°C, the mixture was centrifuged at 4,500 rpm for 15 min and filtered through a 0.45 μm pore filter (Sarstedt, Wexford, Ireland). The filtrate was diluted to 10^−8^ and plaqued on the appropriate media for 24 h at 30°C. These steps were repeated twice on the resulting plaques until a pure phage was obtained.

### Lytic spectrum and adsorption to lactococcal cells

The lytic spectrum of ΦL47 was determined, as for dairy lactococcal phages, by spotting 10 μ L of phage lysate containing 10^7^ PFU/mL onto soft agar seeded with a *L. lactis* strain. Adsorption of phage particles to lactococcal cells was determined as follows: 10 μ L of phage lysate (~10^7^ PFU/mL) was mixed with 2 mL of late exponential cells. Samples were incubated at 30°C for 10 min with shaking, to allow the phage particles to attach. Each sample was centrifuged at 14,000 rpm for 5 min and filtered through a 0.2 μm filter (Sarstedt, Wexford, Ireland). The number of phages in the supernatant was determined by plaque assay and % adsorption was calculated using the formula [(initial titer—titer in supernatant)/ initial titer × 100%].

### Electron microscopy

High titer phage suspensions for visualization of phage particles were prepared using CsCl gradient purification and ammonium acetate concentration. Pure phage samples were obtained using a CsCl gradient of polyethylene glycol (Sigma-Aldrich, Dublin, Ireland) precipitates as described by Sambrook and Russell (2001). For ammonium acetate precipitation, 1 L of fresh lysate was centrifuged at 10,000 rpm at 4°C for 10 min and filter sterilized using a 0.45 μm pore filter (Sarstedt). Phage particles were subsequently precipitated by centrifugation at 20,000 rpm for 1 h at 4°C. The supernatant was removed and the phage pellet re-suspended in 10 mL of ice cold 0.1 M ammonium acetate (Sigma-Aldrich). After pooling of phage samples, a further 10 mL of ammonium acetate was added and the sample centrifuged at 20,000 rpm for 1 h. The supernatant was again removed and the pellet suspended in a final volume of 1 mL ammonium acetate. Negative staining was performed on both phage samples using 2% uranyl acetate on carbon films. Each grid was examined at an 80 kV acceleration voltage using a Tecnai 10 transmission electron microscope (FEI Company, Eindhoven, The Netherlands). Micrograph images were captured using a MegaView 2 CCD-camera (Olympus SIS, Münster, Germany). Phage structure dimensions were determined based on the average of 10–15 measurements.

### Structural analysis of phage proteins

Analysis of structural proteins was performed as described previously (Kelly et al., 2012) using high titer phage suspensions obtained from ammonium acetate concentration. Samples were mixed with 4X sample loading buffer and heated at 95°C prior to loading in a 12% SDS polyacrylamide gel. Protein bands were stained using Coomassie blue staining and excess dye removed using a de-staining solution (40% ethanol, 53% distilled water and 7% acetic acid). Protein size was estimated using a broad range protein ladder (New England Biolabs, Hertfordshire, UK) as a relative molecular weight marker (MWM).

### DNA sequencing, annotation and comparative analysis

Bacteriophage DNA sequencing was performed using the Roche GS FLX+ system to >20× coverage (MWG, Ebersberg, Germany). The quality of the raw assembly reads were visualized and verified using the programme Hawkeye (Amos) (Schatz et al., 2013) and Consed (Gordon, 2003). To verify the genome structure, PCR amplicons were generated with the Platinum Hi-fidelity PCR Supermix (Invitrogen, Life Technologies, Dublin, Ireland) for various regions of the genome and at contig ends, followed by direct sequencing. The final phage genome was assembled using the Phred-Phrap-Consed package (Ewing and Green, 1998; Gordon, 2003). ORFs were predicted using the programs prodigal (Hyatt et al., 2010) and Glimmer (Delcher et al., 1999). Annotation was provided by the RAST annotation software (Aziz et al., 2008) and GAMOLA (Altermann and Klaenhammer, 2003). Genome annotation was verified manually using Artemis (http://www.sanger.ac.uk/resources/software/artemis/) (Rutherford et al., 2000) and detected open reading frames (ORFs) were functionally annotated using BLASTp (Altschul et al., 1990). Conserved domains were detected using InterProScan (http://www.ebi.ac.uk/InterProScan/) and DELTA-BLAST (Domain Enhanced Lookup Time Accelerated BLAST) (Marchler-Bauer et al., 2011). Identification of tRNAs was achieved using the software packages tRNA scan-SE (Lowe and Eddy, 1997) and ARAGORN v1.2.36 (Lowe and Eddy, 1997; Laslett and Canback, 2004). Comparative genome analysis of the *Lactococcus* ΦL47 with its most similar relative, *Lactococcus* Φ949, was performed using the Artemis Comparison Tool (ACT) programme (Carver et al., 2005). The genome sequence of ΦL47 is available from GenBank/EMBL under the accession number KF926093.

## Results and discussion

### Isolation of ΦL47 from sewage

Previous work in our laboratory led to the isolation of a bank of lactococcal strains from non-dairy origins, including grass, vegetable matter and bovine rumen samples. These strains, which included both *L. lactis* ssp. *lactis* and *L. lactis* ssp. *cremoris*, were evaluated for their flavor-forming ability in a mini-Gouda cheese model and were shown to have potential to diversify flavor in this system (D. Cavanagh, unpublished). Future use of these strains in a commercial setting will depend on a number of technological characteristics inherent in the strains, including their sensitivity to phages. The phage sensitivity of the non-dairy isolate bank was assessed using 8 common dairy lactococcal phages. Phage titers in excess of 10^7^ PFU/mL were propagated and tested against the target strain using a spot plate assay performed in triplicate (Table 1). ΦPOO8, ΦbIL170, ΦHP, ΦC2 and ΦKSY1 were incapable of infecting all of the non-dairy isolates tested. However, ΦML3 was capable of infecting strains DPC6855 and DPC6856, isolated from grass and bovine rumen, respectively, while ΦebI infected the strains DPC6854 and DPC6855 but not DPC6856 (Table 1).

**Table 1:** **Phage resistance profile of dairy lactococcal phages to non-dairy *Lactococcus* strains**.

**Strain**		**Phage**							
	**Phage group**	**936**				**c2**			**KSY 1**
	**Origin**	**Φ712**	**ΦPOO8**	**ΦbIL170**	**ΦHP**	**ΦC2**	**ΦebI**	**ΦML3**	**ΦKSY1**
*L. lactis* ssp. *lactis* DPC6853	Corn	−	−	−	−	−	−	−	−
*L. lactis* ssp. *cremoris* DPC6854	Grass	−	−	−	−	−	+	−	−
*L. lactis* ssp. *cremoris* DPC6855	Grass	−	−	−	−	−	+	+	−
*L. lactis* ssp. *cremoris* DPC6856	Rumen	+	−	−	−	−	−	+	−
*L. lactis* ssp. *cremoris* DPC6857	Grass	−	−	−	−	−	−	−	−
*L. lactis* ssp. *cremoris* DPC6858	Grass	−	−	−	−	−	−	−	−
*L. lactis* ssp. *cremoris* DPC6859	Grass	−	−	−	−	−	−	−	−
*L. lactis* ssp. *cremoris* DPC6860	Grass	−	−	−	−	−	−	−	−

Overall, the non-dairy isolates displayed significant levels of insensitivity to the dairy phages. Given the high evolution rate of phages to overcome bacterial defence systems (Sturino and Klaenhammer, 2006), however, it is highly likely that over time, phages would emerge to threaten these bacterial strains. To understand the interaction of these isolates with the phages that infect them, a screening programme was initiated to identify phages specific for some of the non-dairy lactococci. Following 3 enrichment cycles of raw sewage samples with the grass isolate *L. lactis* DPC6860 as a host, phage ΦL47 was isolated. ΦL47 was found to form clear plaques of ~0.6 mm diameter when plaqued on *L. lactis* DPC6860. The formation of clear plaques would suggest that ΦL47 is virulent. Phage titers of 10^7^–10^8^ PFU/mL were recovered after a single propagation.

### Limited host range of *L. lactis* DPC6860

**Figure 1:**
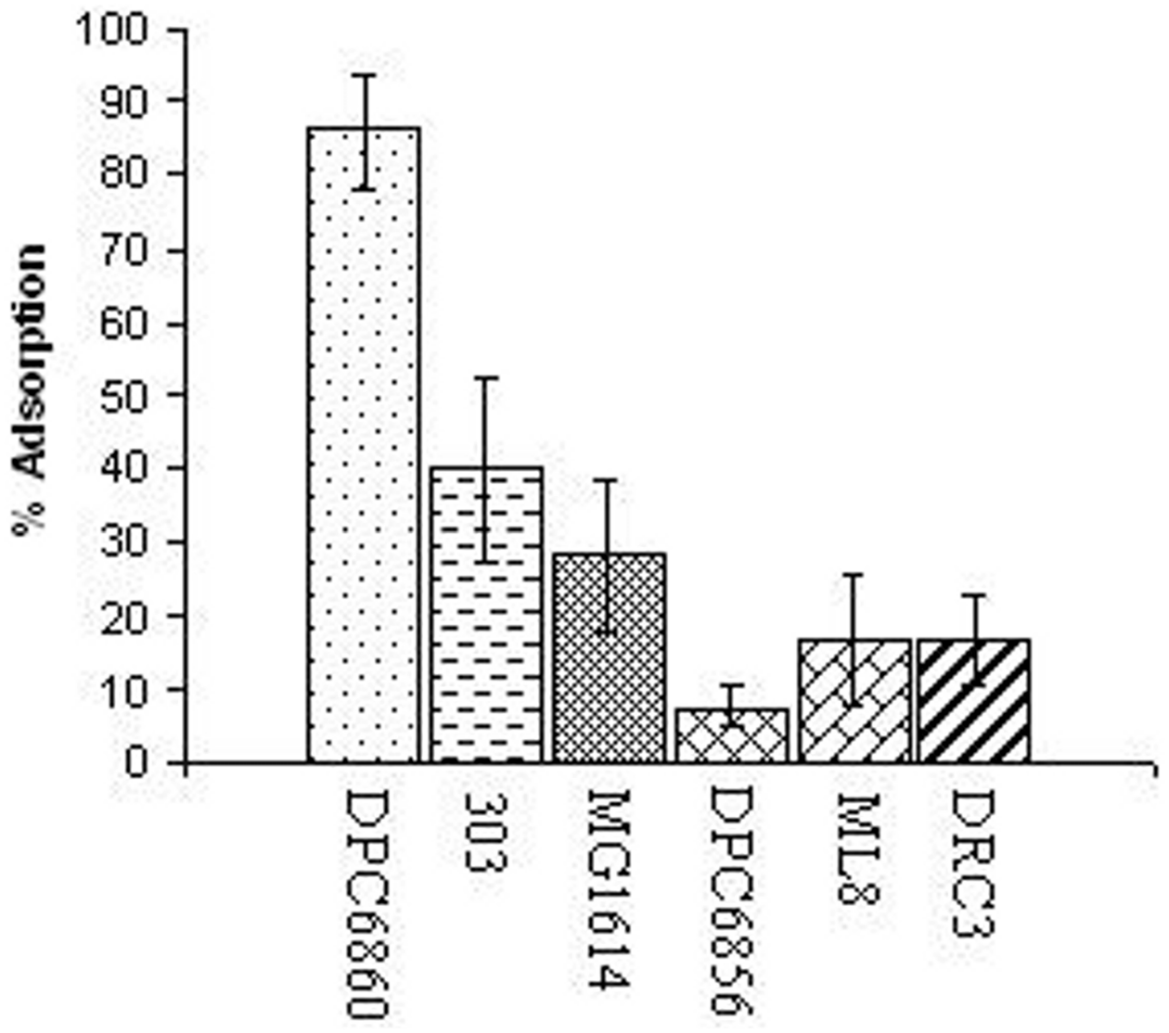
**Relative % adsorption of ΦL47 to *Lactococcus* strains of different environmental niches and subspecies designation**.

In order to determine the host range of ΦL47, a range of lactococcal strains were chosen for analysis including industrial dairy starters, strains of non-dairy origins and a strain from raw milk (Table 2). It was found that ΦL47 is capable of infecting 3 out of 7 non-dairy *Lactococcus* isolates but only 4 out of the 19 dairy strains, corresponding to hosts of 2 phage groups including the 949 group (Table 2). ΦL47 was unable to infect *L. lactis* IL1403, another host strain of Φ949. Contrastingly, although Φ949 was only able to infect 7 of 59 strains of *Lactococcus*, these corresponded to hosts belonging to 5 phage groups (Samson and Moineau, 2010). Levels of adsorption of ΦL47 to *L. lactis* DPC6860 were >85% compared to <40% for other strains tested (Figure 1). However, ΦL47 was capable of lysing strain ML8 but not 303 even though a higher level of phage adsorption was observed in the latter. This would suggest that *L. lactis* 303 possesses a form of anti-phage defence which prevents phage DNA entry or cleaves phage DNA.

**Table 2:** **Host range of *Lactococcus* phage ΦL47**.

**Species**	**Origin/Use**	**Host celllysis**
*L. lactis* ssp. *cremoris* DPC6855	Grass	−
*L. lactis* ssp. *cremoris* DPC6859	Grass	+
*L. lactis* ssp. *cremoris* DPC6854	Grass	−
*L. lactis* ssp. *cremoris* DPC6858	Grass	+
*L. lactis* ssp. *cremoris* DPC6857	Grass	+
*L. lactis* ssp. *cremoris* DPC6856	Rumen	−
*L. lactis* ssp. *lactis* DPC6853	Corn	−
*L. lactis* ssp. *cremoris* HP	Dairy	+
*L. lactis* ssp. *cremoris* IE16	Dairy	−
*L. lactis* ssp. *cremoris* MG1363	Dairy	−
*L. lactis* ssp. *lactis* IL1403	Dairy	−
*L. lactis* SMQ562	Dairy	−
*L. lactis* ssp. *cremoris* UC509	Dairy	−
*L. lactis* ssp. *lactis* biovar diacetylactis F7/2	Dairy	−
*L. lactis* ssp. *lactis* ML8	Cheese	+
*L. lactis* ssp. *cremoris* H88M1	Raw milk	−
*L. lactis* ssp. *lactis* 303	Cheese	−
*L. lactis* HT−2	Dairy	−
*L. lactis* ssp. *cremoris* SK11	Cheese	−
*L. lactis* U53	Dairy	+
*L. lactis* ssp. *lactis* biovar diacetylactis DRC3	Dairy	−
*L. lactis* 83	Dairy	−
*L. lactis* BA1	Dairy	−
*L. lactis* ssp. *lactis* biovar *diacetylactis* 938	Dairy	−
*L. lactis* ssp. *cremoris* 310	Cheese	+
*L. lactis* ssp. *lactis* 229	Cheese	−
*Lactobacillus plantarum*	Grass	−
*Lactobacillus brevis*	Grass	−

### Extended tail fiber of ΦL47

**Figure 2:**
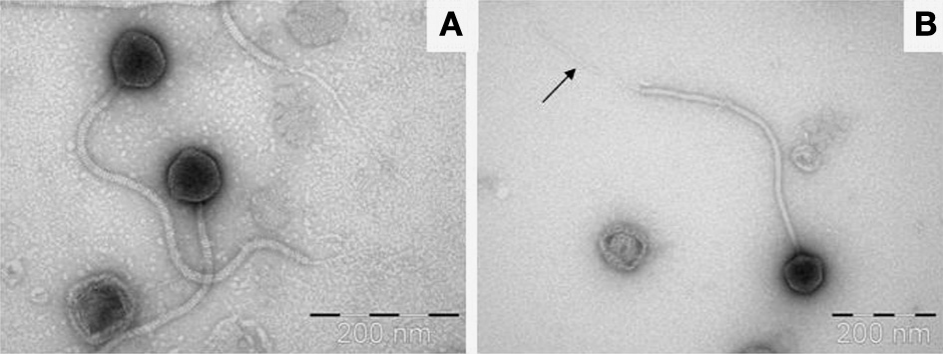
**(A)** Transmission electron micrograph image of ΦL47 negatively stained using 2% uranyl acetate. **(B)** Single view of ΦL47. Arrow shows the elongated tail fiber.

Visualization of phage particles was achieved by transmission electron microscopy performed on samples obtained from both CsCl gradient purification of PEG precipitations and ammonium acetate concentration. Images generated from samples obtained by CsCl gradient showed the majority of phage virions to have disintegrated, with broken tails and empty capsids. Images generated from ammonium acetate preparations displayed intact phage particles which allowed for the estimation of capsid diameter and tail length (Figure 2). It was determined that ΦL47 possesses an icosahedral capsid (diameter 75 nm) and a non-contractile tail (length 480 nm; width 15 nm) indicating that this phage is a member of the family *Siphoviridae* of the class *Caudovirales*. Morphologically, ΦL47 is very similar to the other lactococcal phage, Φ949 which possesses a non-contractile tail of 500 nm in length with an icosahedral capsid of 79 nm in diameter (Jarvis, 1984; Deveau et al., 2006; Samson and Moineau, 2010). However, ΦL47 appears to possess a distinctive tail fiber of 280 nm in length and tail fiber width of <10 nm (Figure 2B). *Siphoviridae* phages frequently possess a tail fiber involved in phage infection, however, a tail fiber of this length has not previously been reported in lactococcal phages (Vegge et al., 2005; Boulanger et al., 2008; Jakutytë et al., 2012). *Enterococcus faecalis* bacteriophage ΦEF24C-P2 was found to possess a long tail fiber which was associated with higher infectivity against bacterial strains than the un-mutated phage which does not have a tail fiber (Uchiyama et al., 2011). Similar to ΦEF24C-P2, an extended tail fiber was found in *Bacteroides fragilis* phage ATCC 51477-BI, which was isolated from wastewater (Hawkins et al., 2008). No bouquet-like arrangement was identified in ΦL47, as demonstrated by phage Φγ of *Bacillus anthracis* which possessed a 63 nm long tail fiber (Schuch and Fischetti, 2006).

### Structural protein analysis

**Figure 3:**
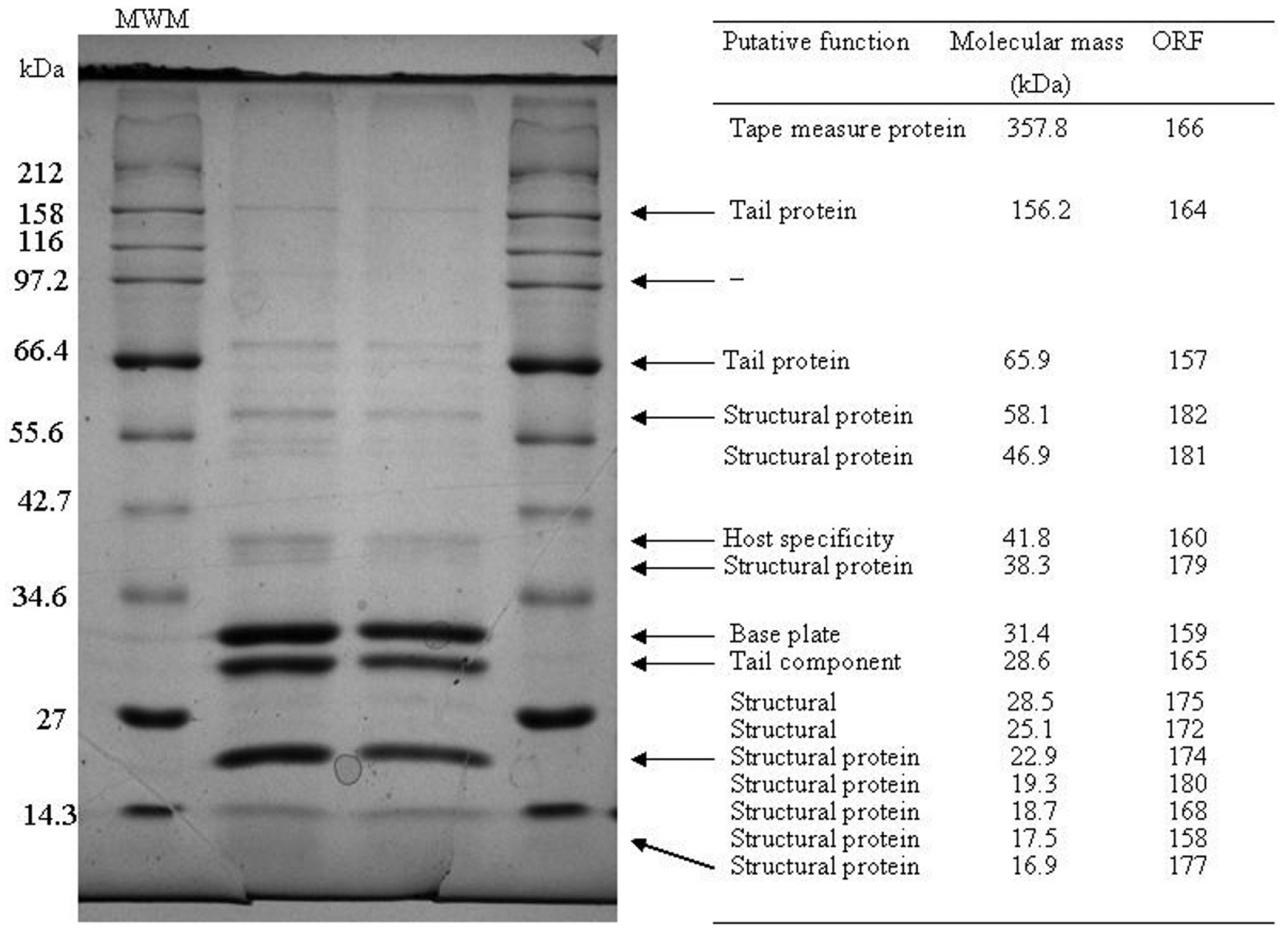
**Analysis of the structural proteins of ΦL47 obtained from ammonium acetate precipitations.** A broad range ladder (New England Biolabs) was used as a relative molecular weight marker (MWM). Molecular masses of structural proteins was predicted from genome sequence data using the Compute pI/ Mw tool (Gasteiger et al., 2005).

SDS-PAGE was employed to isolate the structural proteins of ΦL47 and this led to the identification of 9 individual protein bands (Figure 3). Five proteins of low molecular weight (<30 kDa) identified from genome sequence analysis, were not identified on the gel. These proteins may not have been present at a sufficient concentration to allow for visualization. Molecular masses of proteins estimated by SDS-PAGE corresponded to the estimated molecular weight of structural proteins predicted from genome sequencing. Three major structural proteins were identified from SDS-PAGE, with the largest having a molecular weight of 31.4 kDa and may correspond to a base plate protein (ORF 176). One large minor protein of ~100 kDa was visualized on the gel but did not correspond to any identified ORF. Multiple bands were observed at ~50 kDa and ~30 kDa which could not be assigned to identified ORFs. It is possible that these proteins are proteolytic products of some other phage structural proteins, or they are of bacterial origin. Techniques such as N-terminal sequencing of protein bands would allow for a more definite relationship to be established between genome analyses and SDS-PAGE.

## Analysis of the L47 genome

### General characteristics

To date, all 59 sequenced *L. lactis* phages available in the public domain possess a dsDNA genome and are predominately 15 to 35 kb in size with the exception of the larger genomes of ΦKSY1 (79.2 kb), ΦP087 (60.1 kb), and Φ949 (114.8 kb). Analysis of the ΦL47 genome revealed a dsDNA molecule of 128, 932 bp, making it the largest sequenced lactococcal phage to date. ΦL47 was found to possess a molecular GC content of 32.5% which corresponds to that observed in the similarly sized *Lactococcus* phage, Φ949 (32.7%). The GC content of the host organism, *L. lactis* DPC6860, was calculated as 35.56%. A total of 190 ORFs were identified in the ΦL47 genome, 117 of which shared significant homology with *L. lactis* Φ949 (>50% amino acid identity). The majority of these genes were involved in capsid and tail morphogenesis as well as various hypothetical proteins. Φ949 was isolated from cheese whey in New Zealand over 35 years ago while ΦL47 was isolated from raw sewage indicating how this phage group thrives in their respective environments. Five ORFs were identified that shared no significant identity with proteins in the databases. Similar to the unusual genomic arrangement of Φ949, 4 gene clusters were identified in the ΦL47 genome, with 2 clusters transcribed in the opposite direction. Large inter-genic regions were identified between ORFs 37–38 and ORFs 54–55 which were preceded by a shift in the direction of gene transcription. The 190 ORFs identified in the ΦL47 genome (Table 3) account for 88% of the genome which is the same as that identified in Φ949. No putative function could be assigned to 132 ORFs (69.47%). The predominant start codon (89.71%) was ATG with only 8 ORFs starting with an uncommon start codon (TTG, GTG).

**Table 3:**
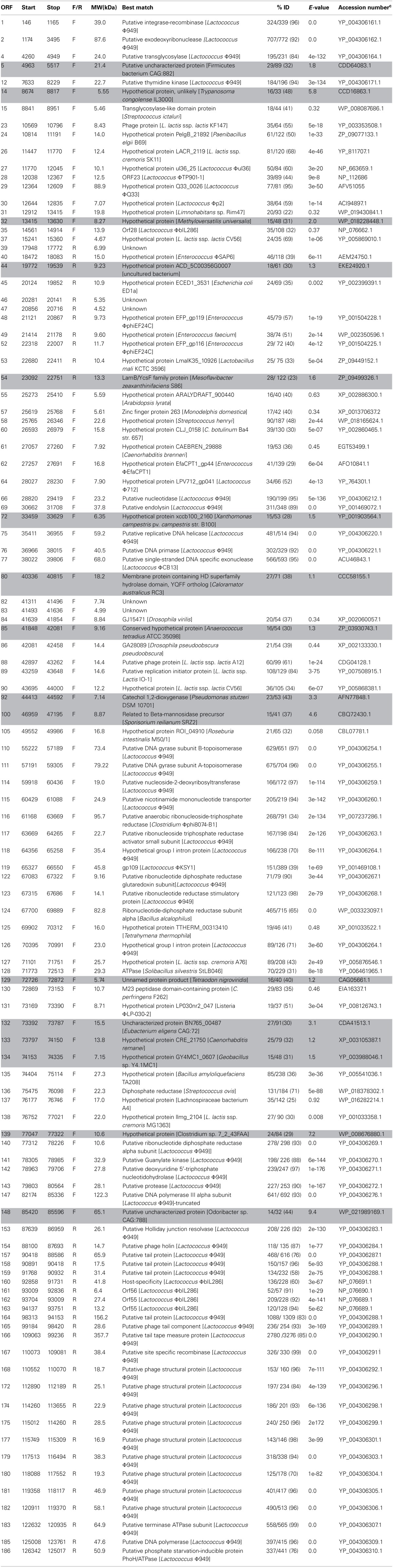
**Predicted open reading frames (ORFs) of ΦL47^a, b^ and predicted database matches**.

*^a^Hypothetical proteins with >60% identity to* Φ*949 have been omitted.*

^b^ORFs shaded gray possess E-values >1.

^c^Accession numbers corresponding to the NCBI database.

Abbreviations: F/ R, Forward/ Reverse; MW, Molecular weight; %ID, % Identity; E-value, Expect-value.

### Tail morphology

**Figure 4:**
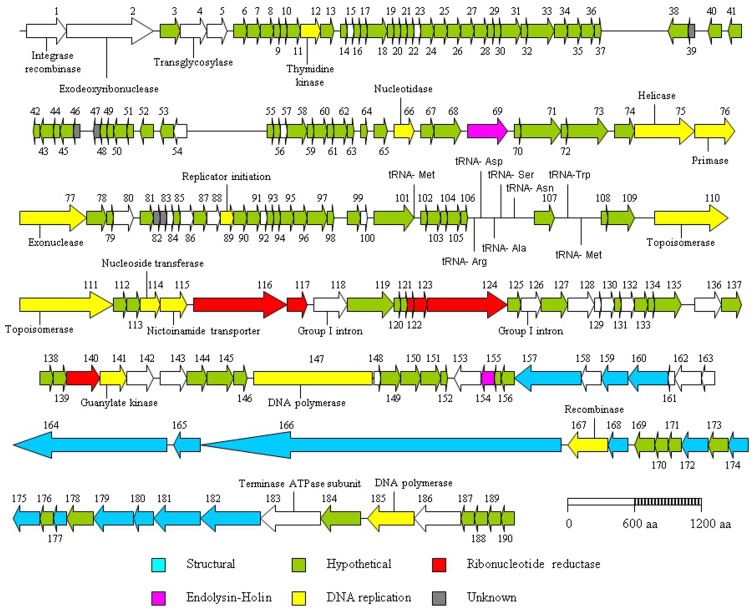
**Genome organization of the lactococcal phage ΦL47.** Each arrow represents an open reading frame (ORF), the orientation of which shows the direction of transcription. The predicted function of each ORF was determined by bioinformatic analyses (Carver et al., 2005).

As stated previously, ΦL47 possessed a long non-contractile tail of 480 nm in addition to a long tail fiber of 280 nm. Both capsid and tail structural genes were encoded from ORFs 157 to 182, and organized in a modular arrangement located downstream from the holin gene (Figure 4). The products of 14 of these ORFs encoded putative structural proteins sharing >70% amino acid identity with Φ949 (Table 3). This is not unusual as the major structural proteins are generally somewhat conserved in related phages (Suyama and Bork, 2001; Ceyssens et al., 2011). In contrast, proteins involved in adsorption to host cells, such as tail fibers, are expected to differ to a larger degree as they are modified to complement with surface receptors of bacterial hosts (Sandmeier et al., 1992; Hatfull, 2002; Desplats and Krisch, 2003; Silhavy et al., 2010). ORFs 160–163, located within the structural module, encode proteins sharing a high percentage identity (>60%) to proteins of ΦbIL286. ORF 159 encodes a protein with a high percentage identity to a putative tail protein of *Lactococcus* Φ949 but also the putative base plate protein of *Lactococcus* ΦP335, and the phage tail assembly protein of *L. lactis* ssp. *cremoris* A76. A conserved domain search of the putative tail protein of Φ949 reveals the presence of a prophage tail super family cl12123 domain which may possibly act as an endopeptidase.

ORF 160 encodes a putative host-specificity protein sharing a 60% identity at the amino acid level with that of the prophage ΦbIL286. This ORF also shares identity to the phage receptor binding protein (35%) and tail host specificity protein (36%) of the plant strain *L. lactis* KF147. ORFs 162 and 163 also indicate homology with ORF 55 of ΦbIL286, both with 89 and 94% identity, respectively. No conserved domains were identified within these two proteins; however, using BLAST_p_, both proteins were found to possess some identity to a putative tail fiber of *Bacillus* ΦphiNIt1. In order to assign a putative function to these ORFs of ΦL47, ORF 55 of ΦbIL286 was analyzed by InterProScan. The C-terminal region of this gene product was predicted to have an immunoglobulin-like domain IPR007110 as found in the major tail subunit of Enterobacteria ΦHK97 (Juhala et al., 2000) and other phages. Immunoglobulin-like domains participate in various functions, including cell-cell recognition and cell-surface receptors. Therefore, it is likely that these genes also form part of the ΦL47 tail.

### Host cell lysis

In dsDNA phages, the combined action of the holin-lysin genes function in the release of new phage particles from an infected cell (Daniel et al., 2007). ORF 69 was found to encode a putative endolysin sharing 89% identity with that of Φ949, which possesses an amidase_2 domain pfam01510. This ORF also shows a high degree of similarity with gp073, sequence ID YP_001469072.1, of the temperate lactococcal phage ΦKSY1 (83% identity). No holin gene was identified in the same locus as the endolysin, however, a putative holin was located remotely from the endolysin (Figure 4). ORF 154 exhibits an 87% identity to the putative phage holin from Φ949, containing a holin LLH superfamily cl09890 domain, a conserved domain of ~100 amino acids found in prophage and phage of Gram-positive bacteria. The arrangement of the holin and endolysin genes in the ΦL47 genome is not typical of lactococcal phages but a similar organization has been reported in Φ949, ΦP087, and Φ1708 (Garneau et al., 2008; Villion et al., 2009).

### DNA replication and nucleotide biosynthesis

The DNA replication module was located downstream from the endolysin gene and upstream from the holin gene. The predicted gene products for ORFs 75 (Helicase), 76 (Primase), 66 (Nucleotidase), 110 and 111 (DNA gyrase) and 114 (Deoxyribosyltransferase) displayed a large degree of homology with Φ949. ORF 89 was identified as a putative replication protein which shares 84% identity with the putative replication initiator protein of *L. lactis* ssp. *lactis* IO-1 (Kato et al., 2012). A DHH domain pfam01368 was identified in ORF 77 which includes the single-stranded DNA exonuclease RecJ. The protein encoded by this ORF exhibits a 95% identity with the single-stranded DNA exonuclease of *Lactococcus* ΦC13, *Lactococcus* ΦCaesusJM1 and *Lactococcus* Φ949. An exonuclease functions to catalyse the cleavage of a single nucleotide from the end of a polynucleotide chain and is involved in DNA repair, recombination and replication (Ceska and Sayers, 1998). No putative endonucleases were identified in the L47 genome, in contrast to two distinct HNH endonucleases in Φ949. Structure specific endonucleases cut at particular DNA structures and in some cases can give a competitive advantage to a given bacteriophage in a mixed infection (Goodrich-Blair and Shub, 1996). It is possible that ΦL47 possesses no HNH endonucleases as it has acquired other characteristics, such as possessing a reduced number of endonuclease recognition sites, that offer an advantage over other bacteriophages. Group I introns are frequently associated with endonucleases (Chevalier and Stoddard, 2001), however, ORFs 118 and 126 were both found to encode this type of intervening sequence (IVS) between ribonucleotide reductase proteins. Previously, two introns were found within the ribonucleotide reductase large subunit of *Staphylococcus aureus* ΦTwort (Landthaler et al., 2002). In *Escherichia coli* ΦT4, the aerobic Ribonucleotide reductase small subunit and the anaerobic ribonucleotide reductase were both found to possess group I introns (Sjöberg et al., 1986; Young et al., 1994). One explanation offered for the retention of introns in genes, such as those involved in ribonucleotide reduction, is that they encode functions replicated in the host organism that can be utilized for phage survival until such time as the insertion element and host environment adapt to one another (Derbyshire and Belfort, 1998).

The acquisition of ribonucleotide reductases (RNRs) is thought to arise from the host genome, to enable the adaptation to certain environmental conditions (Dwivedi et al., 2013). These enzymes function in DNA replication and repair via the conversion of ribonucleotides to deoxyribonucleotides (Dwivedi et al., 2013). The gene products of 7 ORFs were found to encode ribonucleotide di- and tri-phosphate reductases. ORF 116 encodes a putative anaerobic ribonucleoside-triphosphate reductase with a 25% identity to a putative anaerobic ribonucleoside-triphosphate reductase of *Clostridium* Φphi8074-B1. A class III ribonucleotide reductase domain cd01675 was identified within this gene product which uses a FeS cluster and S-adenosylmethionine to generate a glycyl radical. ORF 124 shares a 65% identity with the ribonucleotide-diphosphate reductase subunit alpha of *Bacillus alcalophilus* and contains a class I RNR domain cd01679. In contrast to class III, a class I RNR domain uses a di-iron-tyrosyl radical. The presence of class I and class III RNR genes in ΦL47 conforms with the RNR complement generally found in phages isolated from sewage (Dwivedi et al., 2013). Other ribonucleotide reductases shared a large degree of homology with Φ949 as well as other genes involved in nucleotide transport, modification and degradation.

### tRNA encoding genes in L47 genome

The role of tRNAs in phage genomes is thought to be the encoding of codons that are less frequent in the host genome, thus allowing for the increased expression of phage proteins (Bailly-Bechet et al., 2007). In all, 8 tRNA genes (Table 4) were identified over a small region of the genome (48,524–53,674 bp) in ΦL47, using the tRNA-scan SE and Aragorn software (Lowe and Eddy, 1997; Laslett and Canback, 2004). tRNAs had a %GC content ranging from 39.4 to 50.7. Between ORFs 106 and 107, were positioned tRNA^Arg^, tRNA^Asp^ and tRNA^Ala^ while between ORFs 107 and 108 were tRNA^Asn^, tRNA^Ser^ and tRNA^Trp^. A 58 bp intron, from position 34 to 35, was identified in tRNA^Ser^. Two tRNA^Met^ genes were identified, the first situated between ORFs 101 and 102, and the second between ORFs 107 and 108. The possession of more than one tRNA for a specific amino acid is a phenomenon that has been observed in *Lactobacillus plantarum* bacteriophage ΦLP65 (Chibani-Chennoufi et al., 2004b). The acquisition of tRNA genes from the host is proposed as being a random event and the genes are either retained, via a set of selection mechanisms, or they are lost (Bailly-Bechet et al., 2007). It is conceivable that ΦL47 has acquired two tRNA^Met^ in order to gain a fitness advantage, due to the relatively harsh environment that it inhabits. *In-situ* burst sizes for organisms in a nutrient-poor environment, such as that from which the host was isolated, are generally smaller than when the host is infected in a nutrient rich, chemically defined media (Chibani-Chennoufi et al., 2004a). The retention of particular tRNA genes in phage genomes are thought to correspond to codons that are less abundant in the host genome (Bailly-Bechet et al., 2007) although this was not observed for Φ949 and its host *L. lactis* IL1403 (Samson and Moineau, 2010). Therefore, we may only hypothesize that these tRNA genes are associated with controlling phage protein production and are possibly involved in increasing reproduction rate and reducing latency time (Bailly-Bechet et al., 2007).

**Table 4:** **tRNA arrangement in ΦL47**.

**tRNA**	**Amino acid**	**Anti-codon**	**Size (bp)**	**% GC**	**Start**	**End**
1	Met	CAT	73	47.9	48524	48596
2	Arg	TCT	73	39.7	50343	50415
3	Asp	GTC	75	50.7	51213	51287
4	Ala	CGC	71	50.7	51639	51709
5	Ser	ACT	92	43.5	51967	52116
6	Asn	ATT	76	39.5	52830	52905
7	Trp	CCA	71	39.4	53114	53184
8	Met	CAT	74	55.4	53400	53473

The arrangement of the tRNAs into distinct blocks suggests that they were obtained through separate recombination events with either host DNA, other phages or a combination of both (Weinbauer, 2004). The presence of tRNAs has been found to be particularly common in phages with a larger genome size (Bailly-Bechet et al., 2007). Six tRNA genes were identified in Φ949, the most identified in a lactococcal bacteriophage (Samson and Moineau, 2010). Similarly, in ΦLb338-1 and ΦK, both with a genome larger than 100 kb, 2 and 4 tRNAs were identified, respectively (O'Flaherty et al., 2004; Alemayehu et al., 2009). No tRNA encoding regions were found in 2 recently elucidated lactococcal phage genomes, belonging to a new P335 group, with sizes of ~31 kb (Mahony et al., 2013). However, 5 tRNA genes have been identified in ΦP087, a P087 species, of a smaller genome size (60,074 bp) than Φ949 and ΦL47 (Villion et al., 2009).

### Comparative genome analysis

Numerous ORFs in the L47 genome were found to show identity to putative proteins of other lactococcal phages. ORFs 27–30 encode hypothetical proteins belonging to different lactococcal phages (Table 3). No conserved domains were detected within these ORFs. ORF 119, showing 39% identity with *Lactococcus* ΦKSY1, was found to encode a pfam01139 domain, a conserved domain of the uncharacterized protein family UPF0027. Putative proteins expressed by *Enterococcus* ΦphiEF24C and *Enterococcus faecium* are also found to be similar to those identified in the ΦL47 genome (3 ORFs; >40% identity). Previously, lactococcal ΦP087 was shown to possess genes with a high degree of similarity to structural genes of a prophage of *Enterococcus faecalis* V538 (Villion et al., 2009). Of note, ORF 23 was found to be highly similar to a phage protein of the plant *Lactococcus* strain, KF147. Upstream from the structural genes, ORF 130 encodes a M23 peptidase domain containing protein similar to *Clostridium perfringens* F262 (Nowell et al., 2012). The position of this gene with genes involved in DNA replication, suggests that it is not tail associated as in *Lactococcus* phages Tuc2009 and TP901-1 (Seegers et al., 2004; Stockdale et al., 2013). This peptidase would possibly hydrolyse peptidoglycan via D-Ala–D-Asp endopeptidase activity enabling the penetration of stationary phase lactococcal cells (Samson et al., 2013).

**Figure 5:**
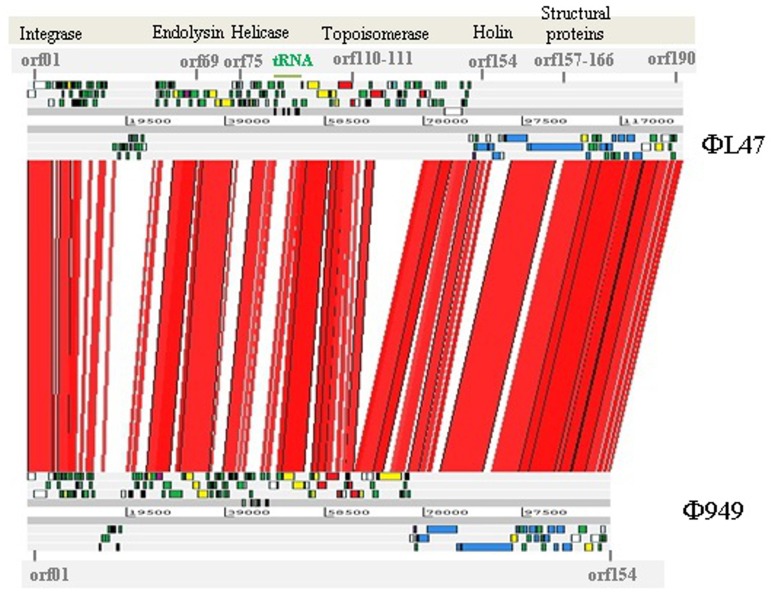
**BLASTn-based alignment (nucleotide identity >90% shown) of the genome sequences of *L. lactis* phage ΦL47 and Φ949 as displayed by the Artemis comparison tool (ACT).** Red lines between genomes indicate orthologs in the same orientation. Coding sequences are color coded as described in Figure 4; structural (blue), hypothetical (green), DNA replication (yellow), ribonucleotide reductase (red), endolysin-holin (mauve), unknown (gray).

Comparative genomic analysis shows the high similarity at a nucleotide level between the ΦL47 and Φ949 phages (Figure 5). In addition, given the close amino acid identity at protein level across the genome (Table 3), suggesting a number of shared functions, it is likely that these phages have shared a common ancestor at some point. Studies of the more common lactococcal phage groups have indicated a large degree of homology between members of the c2 and 936 species while the P335 species displays a polythetic species theory (Deveau et al., 2006). Although much discussion surrounds phage evolution, in relation to virulent phages vertical lines of evolution are believed to be crucial in the development of certain phage families (Brussow and Kutter, 2005) and are particularly evident in dairy phages (Brüssow and Desiere, 2001). However, horizontal genetic exchange also plays a key role in the evolution of phages as exemplified by genetic mosaicism formed from a high occurrence of transfer events (Canchaya et al., 2003). Genetic exchange between bacteria and other phages from distinct niches is hypothesized to be pivotal in the evolution of rare lactococcal phages (Fortier et al., 2006; Chopin et al., 2007; Garneau et al., 2008; Villion et al., 2009). Due to the high degree of sequence homology between these two phages, it is expected that ΦL47 is another member of the 949 group. Analysis of Φ111 revealed this lactococcal phage to possess a genome size of ~134 kb and a long tail of 470 nm (Prevots et al., 1990). However, without comparative genome analysis it can only be speculated that Φ111 is a representative of the 949 group.

## Concluding remarks

To date, numerous studies have investigated dairy lactococcal phages and how they interact with their respective hosts. Industrial *Lactococcus* strains, used in modern cheese production, are thought to have evolved from plant strains (Kelly et al., 2010). ΦL47, isolated from a non-dairy environment, possessed significant similarity to the rare, dairy phage Φ949, with both phages isolated from different environments almost 40 years apart. ΦL47 was found to possess a number of features which differentiate it from Φ949, most notably a long tail fiber, not previously reported in phages of *Lactococcus*. This tail fiber may play an important role in enabling ΦL47 to infect *L. lactis* DPC6860, which was largely resistant to dairy phages, and may account for the persistence of successful virulent phages in the wider environment as observed for ΦQ33 and ΦBM13 (Mahony et al., 2013). Further studies are required to establish the diversity of lactococcal bacteriophages from non-dairy origins and the similarity they may possess with dairy phages of other groups. This information could shed further light on the mechanisms they possess that allow them to thrive in harsher environments and may advance our understanding of host recognition and infection by lactococcal phages.

## Author contributions

Daniel Cavanagh isolated phage, performed biological assays, genome analysis, data analysis and drafted the manuscript. Caitriona M. Guinane performed sequence assembly, analysis and assisted in manuscript drafting. Horst Neve performed transmission electron microscopy of phage particles. R. Paul Ross and Aidan Coffey advised on experimental design. Gerald F. Fitzgerald is a supervisor of this project. Olivia McAuliffe. is a supervisor of this project and assisted in the design of experiments, data analysis and manuscript drafting.

### Conflict of interest statement

The authors declare that the research was conducted in the absence of any commercial or financial relationships that could be construed as a potential conflict of interest.
